# Receipt of humanitarian cash transfers, household food insecurity and the subjective wellbeing of Syrian refugee youth in Jordan

**DOI:** 10.1017/S1368980024002660

**Published:** 2025-01-07

**Authors:** Maia Sieverding, Zeina Jamaluddine

**Affiliations:** 1 Faculty of Health Sciences, Department of Health Promotion and Community Health, American University of Beirut, Beirut, Lebanon; 2 Faculty of Epidemiology and Population Health, London School of Hygiene & Tropical Medicine, London, UK

**Keywords:** Cash transfers, Food security, Youth, Subjective wellbeing, Refugees

## Abstract

**Objective:**

Humanitarian aid, including food aid, has increasingly shifted towards the provision of cash assistance over in-kind benefits. This paper examines whether food security mediates the relationship between receipt of humanitarian cash transfers and subjective wellbeing among Syrian refugee youth in Jordan.

**Design:**

Secondary analysis of the 2020–21 Survey of Young People in Jordan, which is nationally representative of Syrian youth aged 16–30. We employ stepwise model building and structural equation models.

**Setting:**

Jordan.

**Participants:**

Syrian refugee youth aged 16–30 (*n* 1572).

**Results:**

While 92 % of Syrian households with youth received cash transfers from a UN agency, 78 % of households were food insecure using the Food Insecurity Experience Scale. Fifty-one percent of youth suffered from poor wellbeing using the WHO-5 subjective wellbeing scale. Household food insecurity was associated with poorer youth wellbeing. Receiving larger cash transfer amounts was associated with better wellbeing among Syrian youth in unadjusted models. The relationship between receipt of cash transfers and youth wellbeing was not mediated by food security.

**Conclusion:**

We do not find support for the hypothesis that food security is a mediator of the association between cash transfers and subjective wellbeing for this population.

Cash transfers have become an increasingly common modality of assistance in humanitarian crises, including food aid. A growing body of evidence documents the effectiveness of this form of assistance relative to traditional, in-kind aid^(1–3)^. Advocates of cash assistance also point to its operational advantages in terms of transparency, cost-effectiveness and respect for beneficiaries’ needs^(3)^.

The Syrian refugee crisis in the Middle East and North Africa (MENA) region has been at the forefront of the shift from in-kind food aid to unrestricted cash assistance in humanitarian settings. An evaluation of World Food Programme (WFP) assistance for Syrian refugees in Jordan and Lebanon concluded that unrestricted cash was more effective in reducing food insecurity than vouchers that could only be used to purchase food goods from designated shops^(4)^. In both countries, WFP now provides food assistance both through unrestricted cash and food-restricted vouchers^(5,6)^. Evaluations of multipurpose cash assistance for Syrian refugees in Lebanon have found positive impacts on food expenditure^(7)^ and food security^(8)^. In both Lebanon and Jordan, cash assistance has also been found to have positive impacts on a range of child welfare outcomes^(6,9,10)^.

Compared with children, youth, which we follow our data source in defining as those aged 16–30, are a population group that has been less studied in the literature on cash transfers. This may be in part because cash transfer programmes are commonly targeted towards households with children – rather than older youth – or include conditionalities related to child health and school attendance. Yet the transition to adulthood is a key period of life during which cash transfers may support critical investments in health, education and skills development that contribute to long-term socio-economic and health trajectories^(11)^. In the MENA region, youth face substantial challenges in terms of education, school-to-work transition and poor health outcomes^(12,13)^. Among refugee youth, these challenges are compounded by chronic poverty and limited livelihood opportunities that contribute to the adoption of negative coping strategies^(14,15)^.

The potential role of cash transfers in ameliorating the challenges of the transition to adulthood among youth in MENA has been unexplored. In this paper, we aim to address this gap by examining how the receipt of cash transfers is associated with the subjective wellbeing of Syrian refugee youth in Jordan. Specifically, we hypothesise that improved food security mediates the relationship between receipt of cash transfers and improved subjective wellbeing. Psychosocial outcomes such as subjective wellbeing are increasingly recognised as an important outcome of development programmes^(16,17)^. In addition to its intrinsic value as what is arguably the end goal of development, that is, improving individuals’ feelings of happiness and satisfaction with their lives, wellbeing has an instrumental value in fostering better outcomes in areas such as education, health and decision-making^(16)^.

Both cash transfers and food security are theorised to impact subjective wellbeing through psychosocial factors such as self-esteem, reduced stress, reduced family conflict and ability to participate in social networks ^(18–20)^. Receipt of cash transfers is expected to result in immediate changes in income and expenditure, including expenditure on food ^(18)^. The alleviation of income constraints in turn leads to behavioural changes in the household. At this second level, increased household expenditure on food is hypothesised to lead to the consumption of increased quantity and greater diversity of foods and reductions in food insecurity (Fig. 1)^(18)^. This hypothesis is broadly supported by the empirical literature in both development^(18)^ and humanitarian^(1)^ settings. Importantly, intra-household allocation of increased food expenditure may determine who benefits from these hypothesised improvements in food-related outcomes.

**Figure. 1:**
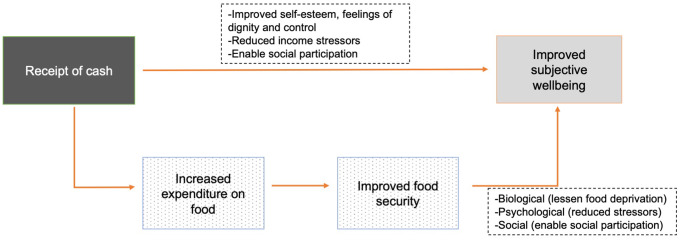
Conceptual framework for the impact of cash transfers on food insecurity and subjective wellbeing.

Food security is in turn strongly associated with improved subjective wellbeing^(21–23)^, including among Arab youth^(19)^. The link between food security and subjective wellbeing may operate through multiple pathways (Fig. 1). On the biological level, food insecurity deteriorates nutritional status through food deprivation. Deteriorated nutritional status is in turn thought to be associated with irritability and depression. On the psychological level, food insecurity leads to both daily and chronic stress and anxiety about food supply. Finally, on the societal level, food insecurity leads to feelings of shame, adoption of negative coping strategies and avoidance of communal activities^(19,21)^.

The psychological and social mechanisms through which food insecurity affects subjective wellbeing are very similar to those hypothesised to link cash transfers and subjective wellbeing directly. Cash transfers may positively impact wellbeing by improving recipients’ self-esteem, feelings of dignity, hopefulness and control, reducing stress and family conflict related to income constraints and enabling greater participation in social events and networks^(16,18,20)^. Recent systematic reviews have concluded that cash transfers have positive effects on the mental health and subjective wellbeing of recipients^(24)^, including among children and youth specifically^(20)^. However, the literature on children and youth is limited and effects are heterogeneous across contexts^(20)^.

There is relatively little literature on the effects of cash transfers on mental health or subjective wellbeing in MENA; neither of the systematic reviews referenced above included any studies from the region. Qualitative studies^(17,25)^ and non-experimental monitoring and evaluation data from humanitarian assistance programmes in the region^(9,10,26)^ find positive associations between cash assistance and mental wellbeing. However, an evaluation of a large-scale conditional cash transfer programme in Egypt found no effects on the prevalence of generalised anxiety disorder among recipient mothers^(27)^. A cross-national survey of vulnerable adolescents during the COVID-19 pandemic, which included Jordan, did not find any associations between household receipt of social assistance and adolescents’ resilience and coping^(28)^. Using the same survey, another study found that, descriptively, in Jordan, the prevalence of poor coping was lower among adolescents in households that received social assistance, but the prevalence of experiencing hunger, anxiety and depression did not differ^(29)^. Our study adds to this emerging literature by examining the relationships between cash transfers, food insecurity and wellbeing among a nationally representative sample of refugee youth in the MENA region in a multivariate framework.

In sum, cash transfers may have both direct impacts on subjective wellbeing and impacts that are mediated through other first- and second-order outcomes, such as food insecurity. A better understanding of the potential role of cash transfers in improving the wellbeing of refugee youth during the transition to adulthood can provide important policy lessons for the delivery of humanitarian assistance. Our specific objectives in this paper are to (1) examine the correlates of receiving different types of humanitarian cash transfers among Syrian households in Jordan that contain youth, (2) quantify the prevalence of poor subjective wellbeing and household-level food insecurity among Syrian refugee youth, (3) analyse the predictors of poor subjective wellbeing among Syrian refugee youth and (4) assess the degree to which the relationship between cash transfers and subjective wellbeing is mediated by food insecurity.

## Methods

### Data: the survey of young people in Jordan

Our analysis is based on the Survey of Young People in Jordan (SYPJ) 2020–21, which was conducted under the sponsorship of UNICEF Jordan^(30)^. The SYPJ is nationally representative of Jordanian and Syrian youth aged 16–30. The survey followed a random, stratified, multi-stage cluster design in which households were sampled and all youth aged 16–30 in the household were invited to participate. Due to the COVID-19 pandemic, data collection with Syrian refugee youth was conducted at different times inside and outside refugee camps. Surveys with Syrian youth residing outside refugee camps, that is, in Jordanian host communities, were conducted in person between August and October 2020. Surveys with Syrian youth living in Jordan’s three official refugee camps for Syrians were conducted by phone in February and March 2021. The total SYPJ Syrian sample consists of 1757 youth in 1069 households.

### Receipt of cash assistance

We focus on three types of cash transfers provided by UN agencies to Syrian refugees in Jordan, namely, WFP food assistance, United Nations High Commissioner for Refugees (UNHCR) multipurpose cash assistance and UNICEF cash assistance for children. While many NGOs also provide cash or voucher-based assistance for refugees, we do not consider these in our analysis as they are often provided for relatively short periods of time^(31)^ and were not common in our empirical data.

WFP assistance has the broadest reach of the three programmes, providing food assistance to approximately 490 000 Syrian refugees as of September 2020^(32)^. Assistance is targeted based on a proxy means test model that assesses vulnerability to food insecurity^(32)^. In 2017, WFP shifted to a ‘choice’ modality of providing food assistance either as unrestricted cash or restricted vouchers^(5,32)^. In host communities, refugee households that choose to receive their benefits as cash may therefore not spend the entire amount on food. Unfortunately, our data do not specify which modality of food assistance households were receiving at the time of the survey, so we treat WFP assistance as a single category.

UNHCR multipurpose cash assistance is provided to refugee households outside of camps that are registered with UNHCR^(33)^. As of August 2021, approximately 30 000 Syrian households received multipurpose cash assistance^(34)^. Eligibility for cash assistance is determined based on a combination of a vulnerability score and the Jordanian poverty line; household size is factored into assistance amounts^(10)^. In 2020, 85 % of Syrian households receiving multipurpose cash assistance reported spending some of the money on food^(33)^.

UNICEF also implements the *Hajati* cash transfer programme targeted at vulnerable children aged 6–15, which is considerably smaller than the other two programmes^(9)^. Our study population was not directly eligible for *Hajati* at the time of data collection because they were aged 16–30. If their households received *Hajati* transfers, it was likely for younger siblings. However, because the cash is unrestricted, it may either be spent directly on food that is split between household members or free up other resources to spend on food and thereby indirectly affect the food security of youth in the household.

Receipt of cash assistance was captured at the household level in the SYPJ. Receipt of multiple forms of assistance was common. We therefore operationalise receipt of assistance as a single categorical variable with the options of (1) no assistance, (2) UNHCR assistance only, (3) WFP assistance only, (4) UNHCR and WFP assistance, (5) UNICEF assistance plus any other type of assistance and (6) all three forms of assistance (UNHCR, WFP and UNICEF).

We conduct a second analysis using the total value of cash assistance per capita in Jordanian dinars (JD), as our key continuous predictor in place of the categorical type(s) of assistance received. For this analysis, the reported monthly value of all forms of cash assistance received was summed for the household and divided by the household size. The distribution of assistance per capita was approximately normal. Dollar equivalents are calculated using the fixed exchange rate of 1·41 USD to the Jordanian Dinar. The data were cleaned for outliers by winsorizing the values of each type of assistance prior to summing.

### Outcome measures

#### Subjective wellbeing

Our key outcome is subjective wellbeing as measured by the WHO-5 wellbeing index. The WHO-5 is grounded in a positive approach to mental health; simply, it seeks to measure emotional states related to happiness^(35)^. The scale consists of five, positively phrased statements about the respondent’s emotional state over the past 2 weeks such as ‘I have felt cheerful and in good spirits’ and ‘I woke up feeling fresh and rested.’ The response items range from ‘all of the time’ (5 points) to ‘at no time’ (0 points). The total score is summed and multiplied by four to generate a scale out of 100, in which 100 represents maximal wellbeing^(36)^. The scale has been widely used internationally and has high validity across sociocultural contexts^(36)^. Subjective wellbeing has also been shown to be more responsive to cash transfers than mental health^(24)^.

Although subjective wellbeing and mental health are distinct, they are closely related. In a number of contexts, a specific cut-off score on the WHO-5 has been validated as a screening indicator for depression^(36)^. This is not the case in the MENA region, so in our descriptive analyses, we follow both the international^(36)^ and small regional^(37,38)^ literatures in categorising WHO-5 scores below 50 as poor subjective wellbeing. In our multivariate analyses, we use the WHO-5 as a continuous outcome.

#### Food insecurity

Food insecurity was assessed using the eight-item Food Insecurity Experience Scale, an experiential measure that includes items related to running out of food, reducing food quality and/or decreasing food quantity due to lack of money or other resources. The Arabic version of the tool has been validated in the MENA region using item response theory measurement models^(39)^. In the SYPJ, household-level food insecurity in the past 12 months was measured. A score was generated by assigning one point to each ‘yes’ response (total scores ranged from 0 to 8). Household food insecurity was then categorised as follows: (0–3) food secure, (4–6) moderately food insecure and (7–8) severely food insecure. It is important to note that because food insecurity was measured at the household and not at the individual level, youth may themselves have higher or lower food insecurity depending on intra-household dynamics of food allocation. However, we cannot assess this with our data.

### Statistical analysis

We conduct descriptive analysis to explore the correlates of receiving different types of assistance at the household level using a *χ* ^2^ test. This analysis focuses on household-level characteristics that may influence eligibility for the different cash transfer programmes. We examine the sex, age, marital status (married *v*. not married; the latter combines the very few households where the head was never married with those where the head was divorced or widowed) and labour force status (out of labour force, employed, unemployed) of the household head. We also examine the total household size, presence of a child under age five (binary), presence of school-aged children (age 6–18; binary) and presence of an elderly member (binary). We do not examine UNHCR registration status because only fifteen Syrian household heads in the SYPJ data were not registered. Finally, we examine camp *v*. non-camp residence and wealth quintile as derived from an asset index. Wealth quintiles were calculated from among Syrian households only because Syrians are overwhelmingly concentrated at the bottom of the wealth distribution as compared with Jordanians. The descriptive characteristics of the households in our sample are presented in the online supplementary material, Supplementary Material Table A1.

We also descriptively examine the sociodemographic correlates of youth subjective wellbeing, based on the categorical outcome of poor *v*. not poor wellbeing, using a *χ* ^2^ test. Covariates were selected *a priori* based on previous literature on the correlates of subjective wellbeing and youth mental health in the MENA region^(19,38,40)^. The individual-level covariates consist of sex, age group (16–17; 18–24; 25–30), education level (less than basic, basic (10th grade), secondary, higher education), current school status (in- *v*. out-of-school), labour force status (out of labour force, employed, unemployed), marital status (coded as ever married *v*. never married since widowhood and divorce were uncommon in this age group) and disability status. For the latter, we use the broad disability definition derived from the UN-Washington Group measure^(41)^. We also include two household-level covariates in our analysis of individual-level youth outcomes: camp *v*. non-camp residence and wealth quintile. The hypothesised relationships between these covariates and youth subjective wellbeing, based on previous literature^(19,38,40)^, are depicted graphically in the online supplementary material, Supplementary Material Figure F1. The descriptive characteristics of youth are presented in the online supplementary material, Supplementary Material Table A2.

To examine the potential mediating role of food security in the association between cash transfers and subjective wellbeing, we use ordinary least squares regression. We first examine the unadjusted (without covariates) and adjusted (with covariates) association between household receipt of cash transfers and youth subjective wellbeing. The covariate set used in the adjusted multivariable models is the same as that used in the descriptive analysis of youth wellbeing. We then add food insecurity into both the unadjusted and adjusted models. We conducted normality tests of residuals and tested for multicollinearity using variance inflation factor analysis. We used a conservative threshold of variance inflation factor < 2·5, which all our covariates met, indicating no significant multicollinearity issues. To understand the effect of each covariate on the estimate and to select the most parsimonious model, we employed a backward stepwise regression approach. This method allowed us to systematically evaluate the impact of each variable on the model. Throughout our analysis, we adhered to conventional standards in social science research, considering results statistically significant at *P* < 0·05. To assess the mediation role of food insecurity, we also conducted an analysis using structural equation models.

Our analytic sample is limited to 1572 youth in 955 households with complete observations on all outcome variables and covariates. Most missing data were at the household level or on variables capturing labour force participation of the household head and youth, which some respondents may have been reluctant to report due to legal restrictions. Youth with and without missing data were not significantly different in terms of sex, age, camp residence, WHO-5 score or food insecurity. Youth with missing data were somewhat more likely to be in households that reported receiving no assistance and less likely to be in households that reported receiving multiple forms of assistance (*P* < 0·001), noting that twenty-two youth were missing data on assistance receipt and forty-five on assistance values. Analysis was conducted using Stata 16 and R studio. All analyses incorporate household or individual-level sample weights as appropriate. Standard errors are clustered at the household level in multivariable analyses.

## Results

### Receipt of cash assistance

Only 7·8 % of Syrian households did not receive any of the three forms of cash assistance (Table 1). The most common form of assistance was WFP, which was the only assistance over half (56·2 %) of households received. Another 14·1 % of households received WFP and UNHCR assistance, and 4·5 % received UNHCR assistance only. UNICEF assistance was relatively uncommon, but 13·5 % of households received all three forms of transfers.

**Table 1.** Receipt of transfers by household characteristics (percentage)

	Household assistance received						
	None	UNHCR*	WFP†	UNHCR + WFP	UNICEF‡ + other	UNHCR + WFP + UNICEF	*P*-value
Overall (%)	7·8	4·5	56·2	14·1	3·9	13·5	
Household characteristics							
Location of residence (%)							0·009
** Non-camp	7·1	3·3	53·0	16·0	4·4	16·2	
** Camp	11·1	10·6	72·2	4·8	1·3	0·0	
Wealth quintile (%)							0·002
** Poorest	8·2	9·5	64·5	7·8	1·2	8·8	
** Q2	16·2	7·1	50·6	19·2	2·2	4·8	
** Q3	3·0	0·9	36·7	19·4	6·4	33·6	
** Q4	4·5	2·7	53·0	14·7	7·9	17·2	
** Richest	6·2	1·6	80·1	8·8	2·1	1·3	
Household head characteristics							
Sex household head (%)							0·094
** Male	7·2	4·7	56·5	11·2	3·8	16·6	
** Female	9·7	3·9	55·0	23·8	4·0	3·5	
Age of household head (%)							0·074
** 20–29	31·3	13·6	43·1	5·4	0·0	6·7	
** 30–39	12·7	1·3	56·9	13·2	3·2	12·8	
** 40–49	2·2	2·9	56·6	23·1	5·3	9·9	
** 50–59	4·4	5·0	57·8	10·7	2·0	20·1	
** 60+	3·4	4·9	60·5	8·4	8·1	14·8	
Marital status of head (%)							0·076
** Married	8·5	4·6	54·6	12·8	4·4	15·2	
** Not married	3·5	4·2	65·9	22·0	0·8	3·7	
Labour force status of head (%)							<0·001
** Out of labour force	5·1	3·2	50·2	14·9	2·3	24·3	
** Unemployed	4·3	7·8	63·9	15·3	5·0	3·7	
** Employed	18·5	3·4	60·6	10·9	6·3	0·4	
Household composition							
Household size (mean)	5·3	8·1	6·2	6·9	7·7	6·9	<0·001
Household child 0–5 (%)							0·174
** No	3·0	5·1	57·3	14·2	3·7	16·6	
** Yes	14·2	3·6	54·7	14·0	4·1	9·4	
Household child 6–18 (%)							0·024
** No	17·4	10·1	65·3	6·7	0·4	0·0	
** Yes	5·9	3·5	54·5	15·5	4·5	16·1	
Household adult 65+ (%)							0·315
** No	8·2	4·5	54·2	14·4	4·2	14·5	
** Yes	1·9	4·7	83·3	10·1	0·0	0·0	
N	74	43	537	135	37	129	955

*P*-values are based on a *χ* ^2^ test.

* United Nations High Commissioner for Refugees.

† World Food Programme.

‡ UNICEF.

There were significant differences in the distribution of assistance types by several household characteristics (Table 1). Receipt of WFP assistance only was considerably higher (72·2 %) in camps than outside (53·0 %). Non-camp residents were more likely to receive UNHCR and WFP or all three forms of assistance (*P* = 0·009). The distribution of assistance was significantly different by wealth quintile (*P* = 0·002), but not in a consistent pattern; it is important to note that receipt of assistance may influence asset acquisition, so this relationship suffers from reverse causality.

Younger (i.e. youth) heads of household more commonly received no assistance and older heads of household all three forms, but the distribution of assistance by age of household head was not significantly different (*P* = 0·074). Households in which the head was out of labour force were most likely to be receiving all three forms of assistance (24·3 %), whereas those in which the household head was employed were more likely to receive no assistance (18·5 %; *P* < 0·001).

Households that received no assistance had the smallest mean size (5·3 persons), whereas those receiving UNHCR assistance only had the largest mean size (8·1; *P* < 0·001). As expected based on eligibility criteria, households with school-aged children were more likely to report receiving UNICEF plus other assistance (4·5 %) or all three forms of assistance (16·1 %; *P* = 0·024).

The mean value of assistance received per month was 26 JD (37 USD) per capita. As expected, households that received multiple transfers reported higher monthly per capita assistance amounts. Households that received UNHCR assistance only received on average 25 JD (35 USD) per capita, those that received WFP assistance 18 JD (25 USD) per capita and those that received UNICEF plus other assistance 26 JD (37 USD). By contrast, households that received UNHCR and WFP assistance reported 42 JD (59 USD) per capita per month and those that received all three transfers 61 JD (86 USD).

### Prevalence and predictors of food insecurity

Only 21·6 % of Syrian households were food secure, 42·4 % were moderately food insecure and 36·0 % were severely food insecure. At the level of youth, this corresponded to 21·7 % food secure, 43·1 % moderately food insecure and 35·2 % severely food insecure. The prevalence of food insecurity was significantly different by location of residence (*P* < 0·001). While 36·7 % of households in refugee camps were food secure, only 18·6 % of those outside refugee camps were food secure. The prevalence of moderate food insecurity was 47·3 % and 41·4 % inside and outside camps, respectively, whereas the prevalence of severe food insecurity was 16·0 % inside camps and 40·0 % outside camps.

### Prevalence of poor subjective wellbeing

There was a substantial burden of poor subjective wellbeing among Syrian refugee youth. Overall, 51·1 % experienced poor subjective wellbeing using the cut-off score of 50 (Table 2). There was no gender difference in experiencing poor wellbeing, but the prevalence of poor wellbeing increased significantly with age. While two-thirds of youth aged 16–17 experienced good wellbeing, two-thirds of those aged 25–30 experienced poor wellbeing (*P* = 0·005).

**Table 2.** Percentage of youth experiencing poor subjective wellbeing by sociodemographic characteristics

	Not poor	Poor	*P*-value
Overall (%)	48·9	51·1	
Individual level characteristics			
Sex (%)			0·625
Male	46·9	53·1	
Female	50·9	49·1	
Age group (%)			0·005
16–17	66·4	33·6	
18–24	41·3	58·7	
25–30	35·7	64·3	
Education (%)			0·266
Less than basic	42·7	57·3	
Basic	52·7	47·3	
Secondary	49·0	51·0	
Higher education	72·9	27·1	
Currently in School (%)			<0·001
Not in school	37·6	62·4	
In school	73·1	26·9	
Labour force (%)			0·005
Out of labour force	46·1	53·9	
Unemployed	27·7	72·3	
Employed	55·1	44·9	
Ever married (%)			0·003
Never married	55·0	45·0	
Ever married	33·9	66·1	
Disability (%)			0·245
No	52·4	47·6	
Yes	40·0	60·0	
Household level characteristics			
Wealth quintile (%)			0·111
Poorest	52·6	47·4	
Q2	31·4	68·6	
Q3	61·3	38·7	
Q4	42·5	57·5	
Richest	55·2	44·8	
Location of residence (%)			0·698
Non-camp	49·3	50·7	
Camp	47·2	52·8	
N	776	796	1572

*P*-values are based on a *χ* ^2^ test. Poor subjective wellbeing is categorised as a WHO-5 score of less than 50.

Although educational attainment was not associated with wellbeing, being out of school – which is also correlated with age – was significantly associated with poor subjective wellbeing (*P* < 0·001). Youth who were unemployed were also considerably more likely to experience poor wellbeing (72·3 %) than those who were out of the labour force (53·9 %) or employed (44·9 %; *P* = 0·005). Being ever married (married or divorced) was significantly associated with poor subjective wellbeing (66·1 %) compared with never married (45·0 %; *P* = 0·003), although this characteristic is again correlated with age. Youth who were disabled were more likely to experience poor wellbeing (60·0 % as compared with 47·6 %), but the result was not statistically significant. Neither household wealth nor camp residence was significantly associated with youth wellbeing.

### Cash transfers and food insecurity as predictors of youth subjective wellbeing

Our descriptive results demonstrate that there is a substantial burden of both food insecurity and poor subjective wellbeing among Syrian refugee youth in Jordan. This is despite the widespread receipt of humanitarian cash transfers, including WFP assistance that is aimed at food purchase. At the same time, there is considerable variation in youths’ experience of poor subjective wellbeing. We turn now to our stepwise model building to test the potential associations between household receipt of cash transfers, food insecurity and subjective wellbeing among youth.

In the unadjusted regression model, only receipt of all three forms of assistance was associated with a higher WHO-5 score (Table 3, column 2; *P* < 0·05). Adding the Food Insecurity Experience Scale into the model did not change this coefficient substantially (Table 3, column 2). Once sociodemographic controls were added to the model, the association between receiving all three forms of assistance and subjective wellbeing became insignificant (Table 3, column 3). When the Food Insecurity Experience Scale was added to the adjusted model, there was again no substantive change in the results for cash transfers (Table 3, column 4). Food insecurity was, however, associated with about an 8-point lower WHO-5 score (*P* < 0·05). The full adjusted model results are provided in the online supplementary material, Supplementary Material Table A3.

**Table 3.** Cash transfers and food insecurity as predictors of subjective wellbeing, ordinary least squares regression model results

	Assistance type								Assistance amount per capita							
	(1)		(2)		(3)		(4)		(5)		(6)		(7)		(8)	
	Unadjusted		Unadjusted		Adjusted		Adjusted		Unadjusted		Unadjusted		Adjusted		Adjusted	
	se	95 % CI	se	95 % CI	se	95 % CI	se	95 % CI	se	95 % CI	se	95 % CI	se	95 % CI	se	95 % CI
Assistance type (ref: none)																
**UNHCR	13·56	(–6·83, 33·94)	14·35	(–5·34, 34·03)	7·90	(–5·66, 21·45)	8·60	(–5·05, 22·25)								
**WFP	9·74	(–5·26, 24·75)	10·29	(–3·59, 24·18)	–1·28	(–11·02, 8·46)	–0·46	(–10·12, 9·21)								
**UNHCR + WFP	10·43	(–4·22, 25·08)	10·20	(–3·13, 23·54)	0·07	(–11·31, 11·45)	–0·27	(–10·96, 10·42)								
**UNICEF + Other	9·80	(–5·85, 25·44)	9·85	(–5·26, 24·97)	1·90	(–9·15, 12·95)	1·98	(–9·17, 13·13)								
**UNHCR + WFP + UNICEF	31·02*	(4·68, 57·36)	30·53*	(3·72, 57·33)	13·67	(–4·92, 32·27)	12·40	(–6·11, 30·92)								
Per capita assistance amount (JD)																
									0·37*	(0·06, 0·68)	0·34*	(0·01, 0·67)	0·19	(–0·03, 0·40)	0·14	(–0·08, 0·35)
FIES (ref: food secure)																
**Moderately food insecure			–8·63	(–18·15, 0·90)			–8·10*	(–15·20, –0·99)			–7·00	(–16·35, 2·34)			–7·85*	(–14·49, –1·22)
**Severely food insecure			–10·53	(–21·23, 0·17)			–8·32*	(–16·56, –0·07)			–8·70	(–21·39, 4·00)			–8·12	(–16·87, 0·63)
**Constant	38·55***	(24·92, 52·18)	45·70***	(32·61, 58·79)	54·62***	(40·33, 68·92)	61·73***	(46·08, 77·38)	41·22***	(33·37, 49·07)	48·04***	(37·07, 59·02)	51·64***	(37·39, 65·89)	60·17***	(43·52, 76·81)
**Observations	1572		1572		1572		1572		1572		1572		1572		1572	

UNHCR, United Nations High Commissioner for Refugees; WFP, World Food Programme; JD, Jordanian dinars; FIES, Food Insecurity Experience Scale.

*P < 0.05; **P < 0.01, ***P < 0.001.

Standard errors clustered at household level; 95 % confidence intervals in brackets.

Adjusted models include controls for sex, age group, education, school status, labour force status, marital status, disability, wealth and camp residence.

In the second panel of Table 3, we present the results using per capita assistance amount rather than types of assistance received as our key predictor. In the unadjusted model, each additional JD of assistance per capita was associated with a 0·37-point increase in youth’s WHO-5 score (*P* < 0·05, Table 3, column 5). As with the categorical assistance outcome, the coefficient of assistance was reduced in the adjusted model and became insignificant (Table 3, column 7). Adding food insecurity did not substantially change the results for either the unadjusted or adjusted model (Table 3, columns 6 and 8).

### Food insecurity as a mediator

**Figure. 2:**
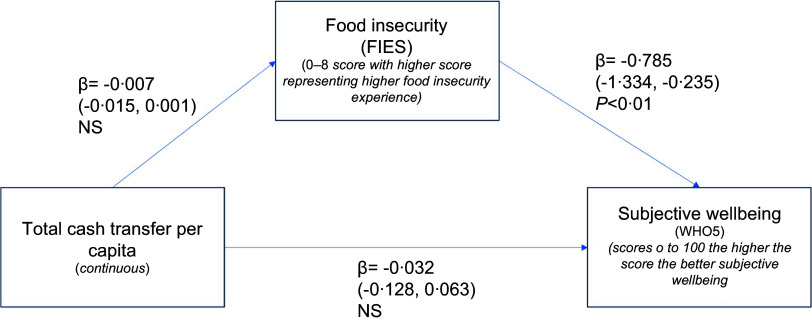
Pathway linking total cash transfer amount per capita, food insecurity and subjective wellbeing, adjusted structural equation model results.

The structural equation model results highlight the relationships between our key variables of cash assistance amount per capita, food insecurity and youth subjective wellbeing (Fig. 2). In the adjusted model, food insecurity experience displayed a negative association with subjective wellbeing (*β* = –0·785, *P* < 0·01), suggesting that higher food insecurity is correlated with lower subjective wellbeing scores. Total cash transfer was not significantly associated with subjective wellbeing or with food insecurity. This indicates that for our study population, food insecurity does not mediate the relationship between cash transfers and subjective wellbeing. The unadjusted model results were substantively equivalent (see online supplementary material, Supplementary Figure F2).

## Discussion

We test the hypothesis that food insecurity plays a mediating role in the relationship between receipt of cash transfers and subjective wellbeing among Syrian refugee youth. This is one of the first studies to examine the association between cash transfers and psychosocial outcomes among youth in the MENA region. In doing so, it contributes to the growing literature on the multisectoral impacts of cash transfers, particularly in humanitarian settings, from a region that is understudied and with a focus on an age group that is seldom addressed in studies on cash transfers.

Our results reveal a substantial burden of food insecurity among Syrian refugee households in Jordan even though 92 % of households received at least one cash transfer from a UN agency and the majority of households were receiving assistance from WFP. These results are consistent with WFP’s own monitoring, which, as of late 2020, about when our data were collected, found that 63 % of refugees in camps and 88 % in host communities were food insecure or vulnerable to food insecurity despite WFP assistance^(32)^. Our prevalence estimates are consistent with those of WFP, despite using a different measure of food insecurity, and likewise demonstrate that the prevalence of food insecurity is higher outside refugee camps. It is unclear whether this is due to receipt of WFP assistance being more common in camps or to other dynamics of income generation and food markets in camp *v*. non-camp settings. Given debates about the impact of encampment policies on refugee wellbeing broadly speaking, this is an important area for further study.

Our results do suggest that, for both camp and non-camp refugee households, and again consistent with WFP^(32)^ and a study of vulnerable adolescents in Jordan^(15,29)^, assistance amounts are not generally sufficient to ensure household food security. Drivers of food insecurity among refugee households were exacerbated by the ongoing COVID-19 pandemic at the time of our study, particularly in camps^(32)^. Contributing factors included loss of WFP school feeding while schools were closed, loss of income opportunities, increased costs of some food items and increased expenditures on some non-food items such as hygiene products^(32)^. Still, food insecurity rates among Syrian refugees were high even prior to the pandemic, ranging between 72 and 80 % among households in host communities and 70–77 % among households in Zaatari camp between 2016 and 2018^(42)^. It is also possible that factors such as the distribution method, duration and consistency of receiving transfers and intra-household dynamics of food allocation play crucial roles in the relationship between cash transfers and food security for this population.

We find a high burden of poor subjective wellbeing among Syrian refugee youth, particularly those aged 18 and above. Comparable population-level estimates for youth in the MENA region are not available using the WHO-5. A similar survey of youth in Egypt found that 6 % of young men and 26 % of young women suffered from poor mental health, which is a substantially lower prevalence than in our study population, but using a different outcome measure^(40)^. While not population-based, other studies among young Syrian refugees in Jordan are consistent in showing a high burden of poor mental health^(29,43,44)^. Mental health concerns among vulnerable young people in Jordan, including refugees, may also have been exacerbated by COVID-19-related stressors and restrictions^(15,28,29)^. Such stressors could have contributed to the high prevalence of poor subjective wellbeing among Syrian youth at the time our data were collected; unfortunately, comparable pre-pandemic estimates are not available. We would argue that the correlations between poor subjective wellbeing and factors such as disability, schooling and labour market status among Syrian refugee youth point to the importance of moving beyond psychosocial support interventions to also addressing the social determinants of refugee mental health.

Given the high burden of poor subjective wellbeing among refugee youth, the question of what role cash transfers may play in supporting positive wellbeing becomes all the more important. Our findings provide indicative evidence suggesting that cash transfers may be associated with improved subjective wellbeing among Syrian refugee youth. Furthermore, it appears to be the amount of the cash transfer that is more predictive of improved wellbeing rather than receipt of a transfer *per se*. In the unadjusted models, it was only receipt of all three transfers (UNHCR, WFP and UNICEF), that is, the combination of transfers with the largest per capita value, which was associated with improved youth subjective wellbeing. Each additional JD of assistance received per capita was also associated with better youth wellbeing. Although the significance of the results was attenuated in the adjusted models, the overall relationship held. In another study that used a binary measure of assistance receipt at the household level, receiving assistance was also not found to be correlated with mental health of vulnerable adolescents in Jordan^(29)^.

Our finding regarding assistance amounts suggests that the mechanism through which receipt of cash transfers may influence refugee youth wellbeing is related to the relief of income constraints (Fig. 1). While receipt of a transfer in and of itself may have psychosocial effects in terms of self-esteem or sense of control, the fact that receipt of smaller value transfers was not associated with better wellbeing suggests that this is not the primary – or a sufficient – mechanism in our study context. This may be particularly true because the humanitarian assistance captured in our study is not targeted towards youth specifically and resulting spending is unlikely to be directly controlled by youth. Rather, cash transfers provide household income that benefits youth directly or indirectly in a context of widespread poverty.

We do not, however, find evidence for our hypothesis that food insecurity is a mediator between cash transfers and subjective wellbeing among the Syrian refugee youth population. Inclusion of food insecurity in our multivariable models did not change the association between receipt of cash assistance and the results of the structural equation model were insignificant. This suggests that, while cash assistance may be associated with youth wellbeing through the mechanism of reducing income constraints, expenditure on food is not a key component of the relationship. Possible explanations for this finding include the insufficiency of assistance amounts to ensure food security or other aspects of food security such as the quality (rather than quantity) of food purchased and consumed. It is also possible that for youth, other types of household expenditure, for example, on education or social activities, or reduced household stress due to increased income, are more important mechanisms for improved subjective wellbeing.

Several important limitations to our analysis should be kept in mind when interpreting these results. First, because food insecurity is measured at the household level, individual youth may experience higher or lower levels of food insecurity depending on intra-household allocation of food. This is a common limitation of the literature on cash transfers in humanitarian settings^(3)^. Second, our analysis cannot be interpreted as causal. We are limited by the lack of data on household food security prior to the start of receiving assistance, which means that we cannot address the endogeneity between food security status and receipt of cash transfers. Third, our measures of receipt of assistance are self-reported by refugee households and are therefore subject to reporting and recall bias as compared with administrative data. However, the forms and amounts of assistance received by households in our data are generally consistent with targeting criteria and assistance amounts as reported by programme documentation.

These limitations notwithstanding, our findings do point to some association between receipt of transfers and subjective wellbeing among Syrian refugee youth. Subjective wellbeing among youth is an important outcome for future evaluation studies of humanitarian cash transfers, including programmes that target children but for whom the mechanisms between receipt of transfers and wellbeing may be similar. Longitudinal data are critical to assessing more rigorously the potential role of cash transfers in addressing the many challenges of the transition to adulthood in the MENA region and to designing the corresponding age-sensitive social protection programmes.

## Supporting information

Sieverding and Jamaluddine supplementary material Sieverding and Jamaluddine supplementary material

## Supplementary material

For supplementary material accompanying this paper, visit https://doi.org/10.1017/S1368980024002660.
