# Supplementary material for: Receipt of humanitarian cash transfers, household food insecurity and the subjective wellbeing of Syrian refugee youth in Jordan
Source: Public Health Nutr. 2025 Jan 7;28(1):e25. doi: 10.1017/S1368980024002660 (PMC11822618; doi:10.1017/S1368980024002660)

**Supplementary Material**

**Table A1: Characteristics of households**

|  | **Col %** | **N** |
| --- | --- | --- |
| **Location of residence (%)** |  |  |
| Non-camp | 83.4 | 511 |
| Camp | 16.6 | 444 |
| **Wealth quintile (%)** |  |  |
| Poorest | 21.7 | 404 |
| Q2 | 21.3 | 169 |
| Q3 | 21.8 | 124 |
| Q4 | 17.8 | 146 |
| Richest | 17.5 | 112 |
| **Sex of household head (%)** |  |  |
| Male | 76.7 | 727 |
| Female | 23.3 | 228 |
| **Age of household head (%)** |  |  |
| 20-29 | 9.8 | 102 |
| 30-39 | 18.5 | 159 |
| 40-49 | 30.9 | 304 |
| 50-59 | 26.8 | 277 |
| 60+ | 14.1 | 113 |
| **Marital status of head (%)** |  |  |
| Married | 85.9 | 829 |
| Not married | 14.1 | 126 |
| **Labour force status of household head (%)** | |  |
| Out of labour force | 51.1 | 455 |
| Unemployed | 27.4 | 282 |
| Employed | 21.5 | 218 |
| **Household size (mean)** | 6.2 | 955 |
| **Household child 0-5 (%)** |  |  |
| No | 57.5 | 491 |
| Yes | 42.5 | 464 |
| **Household child 6-18 (%)** |  |  |
| No | 15.9 | 155 |
| Yes | 84.1 | 800 |
| **Household adult 65+ (%)** |  |  |
| No | 93.2 | 899 |
| Yes | 6.8 | 56 |
| **Total** | 100 | 955 |

**Table A2: Characteristics of youth, by sex**

|  | **Men** | **Women** | **Total** | **N** |
| --- | --- | --- | --- | --- |
| **Age group (%)** |  |  |  |  |
| 16-17 | 36.8 | 31.5 | 34.2 | 336 |
| 18-24 | 46.0 | 48.7 | 47.3 | 881 |
| 25-30 | 17.2 | 19.8 | 18.5 | 355 |
| **Education (%)** |  |  |  |  |
| Less than basic | 41.5 | 34.1 | 37.8 | 582 |
| Basic | 36.8 | 32.9 | 34.9 | 522 |
| Secondary | 18.9 | 27.7 | 23.2 | 394 |
| Higher education | 2.8 | 5.3 | 4.0 | 74 |
| **Currently in School (%)** |  |  |  |  |
| Not in school | 65.3 | 71.5 | 68.4 | 1,184 |
| In school | 34.7 | 28.5 | 31.6 | 388 |
| **Labour force (%)** |  |  |  |  |
| Out of labour force | 28.2 | 8.7 | 18.6 | 330 |
| Unemployed | 22.0 | 11.1 | 16.6 | 346 |
| Employed | 49.8 | 80.2 | 64.8 | 896 |
| **Ever married (%)** |  |  |  |  |
| Never married | 82.0 | 59.5 | 70.9 | 1,002 |
| Ever married | 18.0 | 40.5 | 29.1 | 570 |
| **Disability (%)** |  |  |  |  |
| No | 66.5 | 76.4 | 71.4 | 1,201 |
| Yes | 33.5 | 23.6 | 28.6 | 371 |
| **Wealth quintile (%)** |  |  |  |  |
| Poorest | 26.3 | 29.5 | 27.9 | 675 |
| Q2 | 24.9 | 17.8 | 21.4 | 265 |
| Q3 | 21.5 | 21.4 | 21.5 | 197 |
| Q4 | 15.4 | 13.6 | 14.5 | 247 |
| Richest | 12.0 | 17.6 | 14.8 | 188 |
| **Location of residence (%)** |  |  |  |  |
| Non-camp | 75.8 | 82.3 | 79.0 | 844 |
| Camp | 24.2 | 17.7 | 21.0 | 728 |
| **Total** | 100.0 | 100.0 | 100.0 | 1,572 |

**Supplementary Figure F1: Hypothesized relationships between the covariates and subjective wellbeing**

**
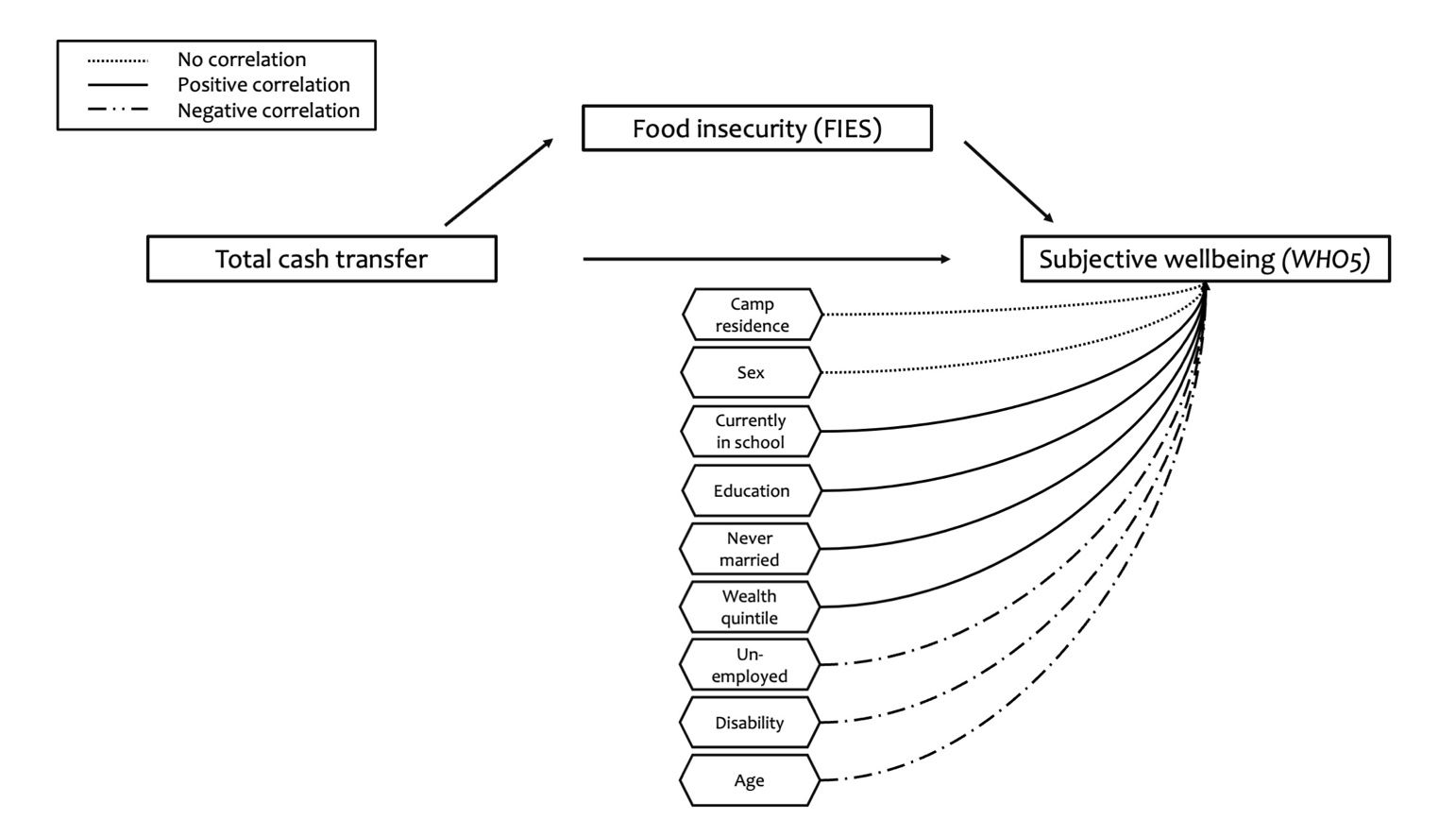
**

**Table A3: Full results for adjusted OLS regression models of cash transfers and food insecurity as predictors of subjective wellbeing**

|  | **Assistance type** | | | | **Assistance amount per capita** | | | |
| --- | --- | --- | --- | --- | --- | --- | --- | --- |
|  | **(1)** | **(2)** | **(3)** | **(4)** | **(5)** | **(6)** | **(7)** | **(8)** |
|  | **Unadjusted** | **Unadjusted** | **Adjusted** | **Adjusted** | **Unadjusted** | **Unadjusted** | **Adjusted** | **Adjusted** |
| **Assistance type (ref: none)** |  |  |  |  |  |  |  |  |
| UNHCR | 13.56 | 14.35 | 7.90 | 8.60 |  |  |  |  |
|  | [-6.83 - 33.94] | [-5.34 - 34.03] | [-5.66 - 21.45] | [-5.05 - 22.25] |  |  |  |  |
| WFP | 9.74 | 10.29 | -1.28 | -0.46 |  |  |  |  |
|  | [-5.26 - 24.75] | [-3.59 - 24.18] | [-11.02 - 8.46] | [-10.12 - 9.21] |  |  |  |  |
| UNHCR + WFP | 10.43 | 10.20 | 0.07 | -0.27 |  |  |  |  |
|  | [-4.22 - 25.08] | [-3.13 - 23.54] | [-11.31 - 11.45] | [-10.96 - 10.42] |  |  |  |  |
| UNICEF + Other | 9.80 | 9.85 | 1.90 | 1.98 |  |  |  |  |
|  | [-5.85 - 25.44] | [-5.26 - 24.97] | [-9.15 - 12.95] | [-9.17 - 13.13] |  |  |  |  |
| UNHCR + WFP + UNICEF | 31.02* | 30.53* | 13.67 | 12.40 |  |  |  |  |
|  | [4.68 - 57.36] | [3.72 - 57.33] | [-4.92 - 32.27] | [-6.11 - 30.92] |  |  |  |  |
| **Assistance amount per capita (JD)** | |  |  |  | 0.37* | 0.34* | 0.19 | 0.14 |
|  |  |  |  |  | [0.06 - 0.68] | [0.01 - 0.67] | [-0.03 - 0.40] | [-0.08 - 0.35] |
| **FIES (ref: food secure)** |  |  |  |  |  |  |  |  |
| Moderately food insecure |  | -8.63 |  | -8.10* |  | -7.00 |  | -7.85* |
|  |  | [-18.15 - 0.90] |  | [-15.20 - -0.99] |  | [-16.35 - 2.34] |  | [-14.49 - -1.22] |
| Severely food insecure |  | -10.53 |  | -8.32* |  | -8.70 |  | -8.12 |
|  |  | [-21.23 - 0.17] |  | [-16.56 - -0.07] |  | [-21.39 - 4.00] |  | [-16.87 - 0.63] |
|  |  |  |  |  |  |  |  |  |
| **Sex (ref: male)** |  |  |  |  |  |  |  |  |
| Female |  |  | 1.84 | 1.28 |  |  | 1.37 | 0.75 |
|  |  |  | [-6.54 - 10.23] | [-6.80 - 9.37] |  |  | [-7.09 - 9.83] | [-7.43 - 8.94] |
| **Age group (ref: 16-17)** |  |  |  |  |  |  |  |  |
| 18-24 |  |  | -7.62 | -8.06 |  |  | -6.63 | -7.21 |
|  |  |  | [-17.18 - 1.94] | [-17.47 - 1.35] |  |  | [-16.65 - 3.39] | [-17.07 - 2.65] |
| 25-30 |  |  | -9.88 | -9.37 |  |  | -8.50 | -8.20 |
|  |  |  | [-23.87 - 4.12] | [-23.12 - 4.37] |  |  | [-22.80 - 5.80] | [-22.30 - 5.90] |
| **Education (ref: less than basic)** | |  |  |  |  |  |  |  |
| Basic |  |  | 2.93 | 3.72 |  |  | 2.69 | 3.54 |
|  |  |  | [-5.87 - 11.73] | [-4.79 - 12.24] |  |  | [-6.11 - 11.49] | [-4.93 - 12.02] |
| Secondary |  |  | -3.49 | -3.17 |  |  | -4.67 | -4.27 |
|  |  |  | [-12.44 - 5.45] | [-11.56 - 5.22] |  |  | [-13.79 - 4.46] | [-12.96 - 4.43] |
| Higher education |  |  | 11.51 | 10.65 |  |  | 11.23 | 10.32 |
|  |  |  | [-5.89 - 28.90] | [-7.27 - 28.56] |  |  | [-6.53 - 28.99] | [-7.99 - 28.62] |
| **Currently in school (ref: no)** | |  |  |  |  |  |  |  |
| In school |  |  | 11.41* | 10.79* |  |  | 12.72* | 12.16* |
|  |  |  | [0.71 - 22.10] | [0.19 - 21.39] |  |  | [0.69 - 24.76] | [0.21 - 24.11] |
| **Labour force status (ref: out of labour force)** | |  |  |  |  |  |  |  |
| Unemployed |  |  | -5.07 | -3.63 |  |  | -6.06 | -4.43 |
|  |  |  | [-12.97 - 2.83] | [-11.74 - 4.47] |  |  | [-13.50 - 1.37] | [-12.16 - 3.30] |
| Employed |  |  | -0.29 | 0.86 |  |  | -0.49 | 0.76 |
|  |  |  | [-9.09 - 8.51] | [-7.70 - 9.43] |  |  | [-9.54 - 8.56] | [-7.99 - 9.51] |
| **Ever married (ref: never married)** | |  |  |  |  |  |  |  |
| Ever married |  |  | -3.34 | -2.91 |  |  | -2.11 | -1.95 |
|  |  |  | [-12.16 - 5.48] | [-11.32 - 5.50] |  |  | [-11.05 - 6.82] | [-10.57 - 6.66] |
| **Disability (ref: no)** |  |  |  |  |  |  |  |  |
| Yes |  |  | -9.57* | -9.60* |  |  | -7.44 | -7.66 |
|  |  |  | [-17.81 - -1.32] | [-17.46 - -1.73] |  |  | [-15.70 - 0.83] | [-15.64 - 0.32] |
| **Wealth quintile (ref: poorest)** | |  |  |  |  |  |  |  |
| Q2 |  |  | -9.65 | -11.21* |  |  | -11.54* | -13.06* |
|  |  |  | [-19.62 - 0.31] | [-21.28 - -1.13] |  |  | [-21.40 - -1.67] | [-22.99 - -3.12] |
| Q3 |  |  | 6.59 | 5.29 |  |  | 5.57 | 4.41 |
|  |  |  | [-4.59 - 17.78] | [-6.48 - 17.07] |  |  | [-6.39 - 17.53] | [-8.16 - 16.97] |
| Q4 |  |  | -6.54 | -8.21 |  |  | -7.55 | -9.21 |
|  |  |  | [-20.29 - 7.21] | [-21.93 - 5.51] |  |  | [-20.22 - 5.12] | [-21.87 - 3.45] |
| Richest |  |  | 3.34 | 0.54 |  |  | 1.13 | -1.57 |
|  |  |  | [-6.31 - 12.99] | [-10.01 - 11.09] |  |  | [-8.30 - 10.56] | [-11.79 - 8.64] |
| **Location of residence (ref: non-camp)** | |  |  |  |  |  |  |  |
| Camp |  |  | 2.35 | -0.35 |  |  | 0.93 | -1.65 |
|  |  |  | [-5.26 - 9.96] | [-9.24 - 8.55] |  |  | [-6.23 - 8.08] | [-9.99 - 6.70] |
| **Constant** | 38.55*** | 45.70*** | 54.62*** | 61.73*** | 41.22*** | 48.04*** | 51.64*** | 60.17*** |
|  | [24.92 - 52.18] | [32.61 - 58.79] | [40.33 - 68.92] | [46.08 - 77.38] | [33.37 - 49.07] | [37.07 - 59.02] | [37.39 - 65.89] | [43.52 - 76.81] |
| Observations | 1,572 | 1,572 | 1,572 | 1,572 | 1,572 | 1,572 | 1,572 | 1,572 |
| *Notes: *p<0.05; **p<0.01, ***p<0.001* | | | | | | | | |
| *Standard errors clustered at household level; 95% confidence intervals in brackets* | | | | | | | | |

**Supplementary Figure F2: Pathway linking total cash transfer amount per capita, food insecurity and subjective wellbeing, unadjusted structural equation model results**


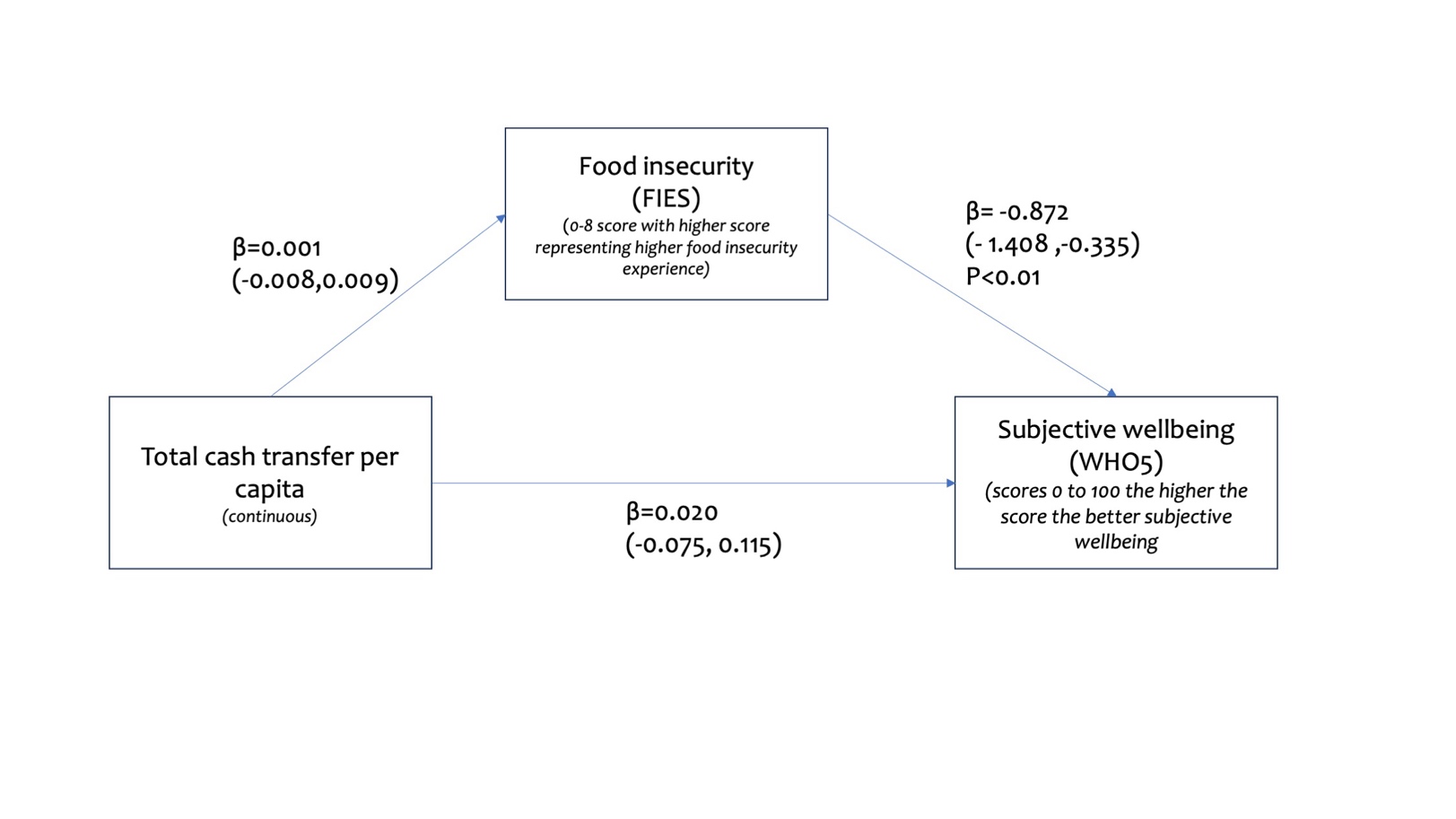

Supplement: Sieverding and Jamaluddine supplementary material [file S1368980024002660sup001.docx]
